# Supplementary material for: Deep-learning structure elucidation from single-mutant deep mutational scanning
Source: Nat Commun. 2025 Jul 25;16:6874. doi: 10.1038/s41467-025-62261-4 (PMC12297490; doi:10.1038/s41467-025-62261-4)
Supplement: Supplementary file 1 — Supplementary Information [file 41467_2025_62261_MOESM1_ESM.pdf]

Supplementary Information

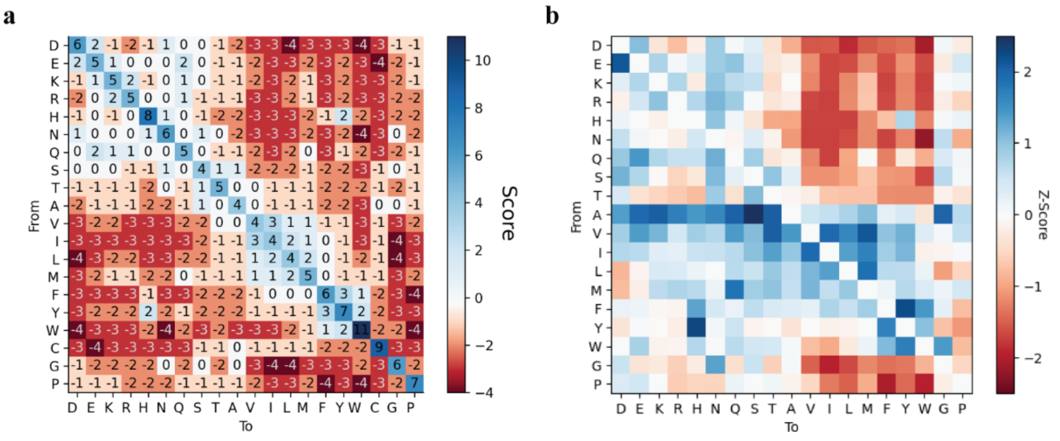

Supplementary Figure 1) a) BLOSUM62 scoring matrix. High and low BLOSUM scores indicate whether a mutation is likely conservative or nonconservative, respectively. b) Z-scores of all residue substitution types between normalized BLOSUM62 scores and mutational correlations derived from comparing burial extent and ΔΔGs of the mega-scale dataset. A high Z-score means a particular mutation is more likely to be detrimental according to our analysis. It corresponds to a higher BLOSUM score but exhibits a stronger mutation correlation. A low Z-score means a particular mutation is more likely to be detrimental according to BLOSUM. It corresponds to a lower BLOSUM score but has a weaker mutation correlation.

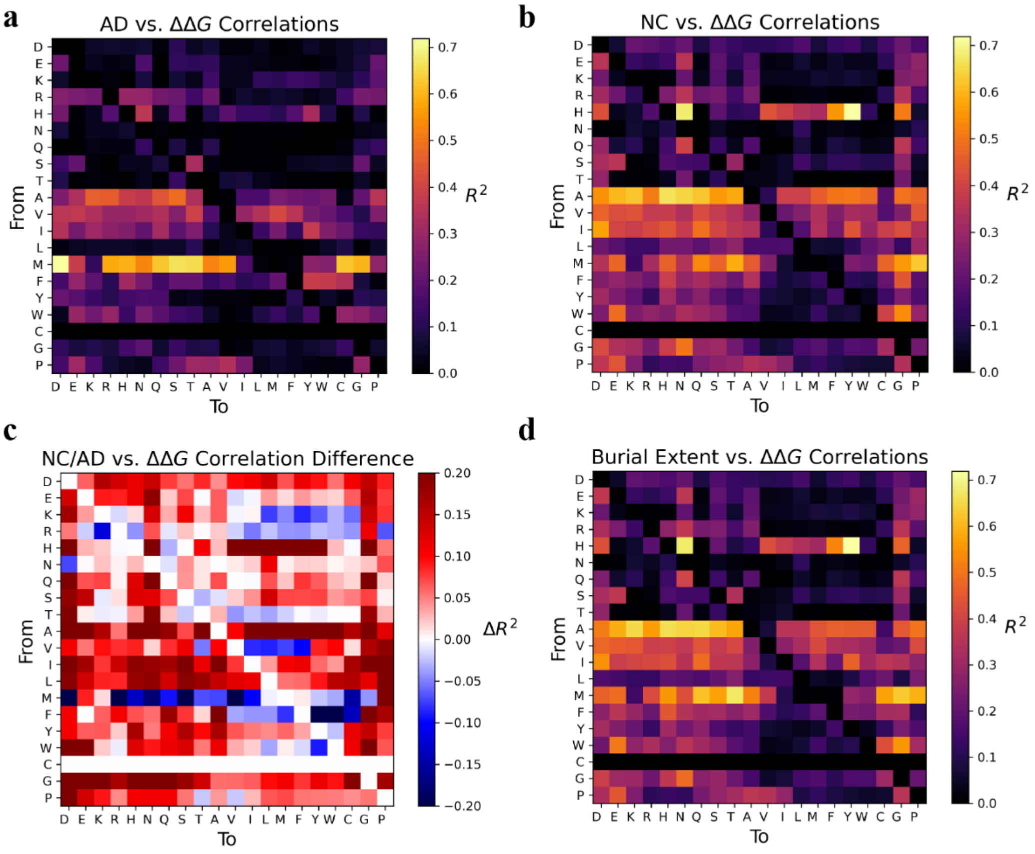

Supplementary Figure 2) Heat maps depicting the correlation between changes in protein thermodynamic stabilities (ΔΔGs) and solubility metrics, atomic depth (AD) and neighbor count (NC), for individual mutational types across the first mega-scale subset (35 proteins). a) Comparing ΔΔGs to native residue atomic depth. b) Comparing ΔΔGs to native neighbor count. c) Differences in correlation coefficients between atomic depth and neighbor count. d) Comparing ΔΔGs to burial extent (defined as weighted average of both atomic depth and neighbor count).

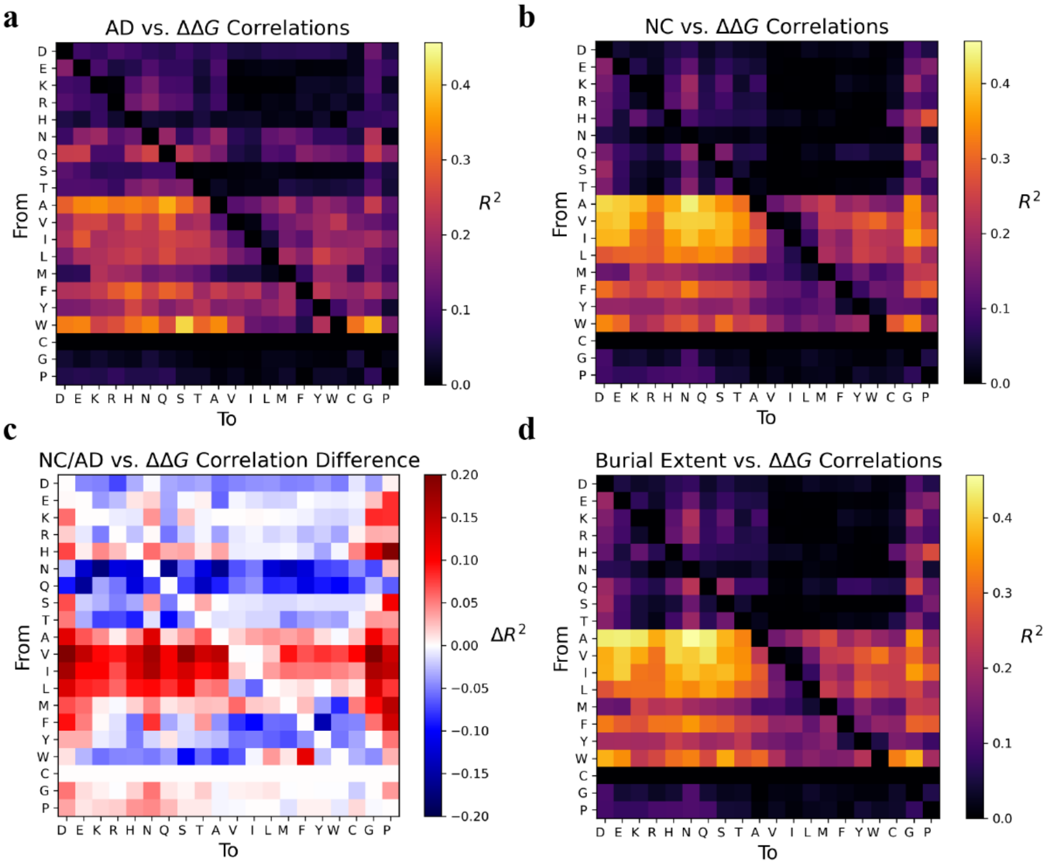

Supplementary Figure 3) Heat maps depicting the correlation between changes in protein thermodynamic stabilities (ΔΔGs) and solubility metrics, atomic depth (AD) and neighbor count (NC), for individual mutational types across the second mega-scale subset (140 proteins). a) Comparing ΔΔGs to native residue atomic depth. b) Comparing ΔΔGs to native neighbor count. c) Differences in correlation coefficients between atomic depth and neighbor count. d) Comparing ΔΔGs to burial extent (defined as weighted average of both atomic depth and neighbor count).

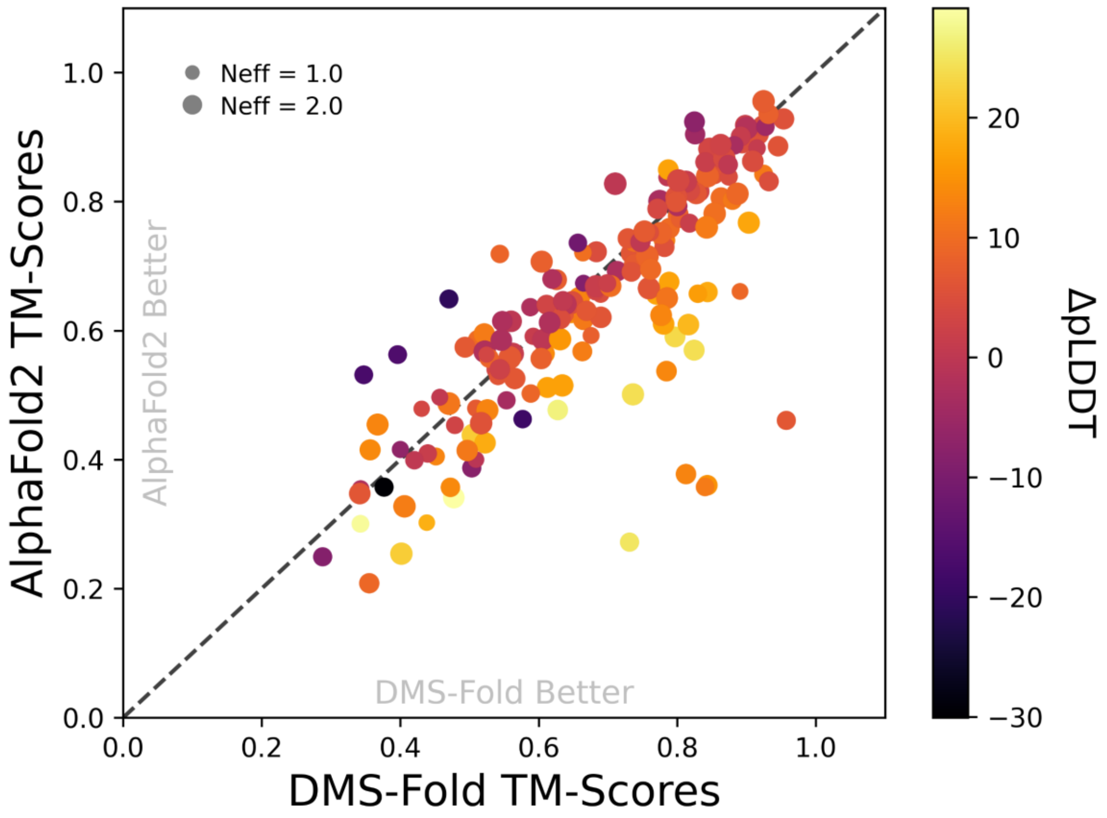

Supplementary Figure 4) Performance of DMS-Fold trained using THPLM protein thermodynamic stabilities (ΔΔGs) on the mega-scale targets with experimental ΔΔGs. a) Template modeling score (TM-Score) comparison of predictions from DMS-Fold and AlphaFold2 using a size-dependent number of nonredundant sequences (N_{eff}). Size of each marker represents the N_{eff} used for MSA subsampling. Color represents the change in network confidence, pLDDT, between DMS-Fold and AlphaFold2.

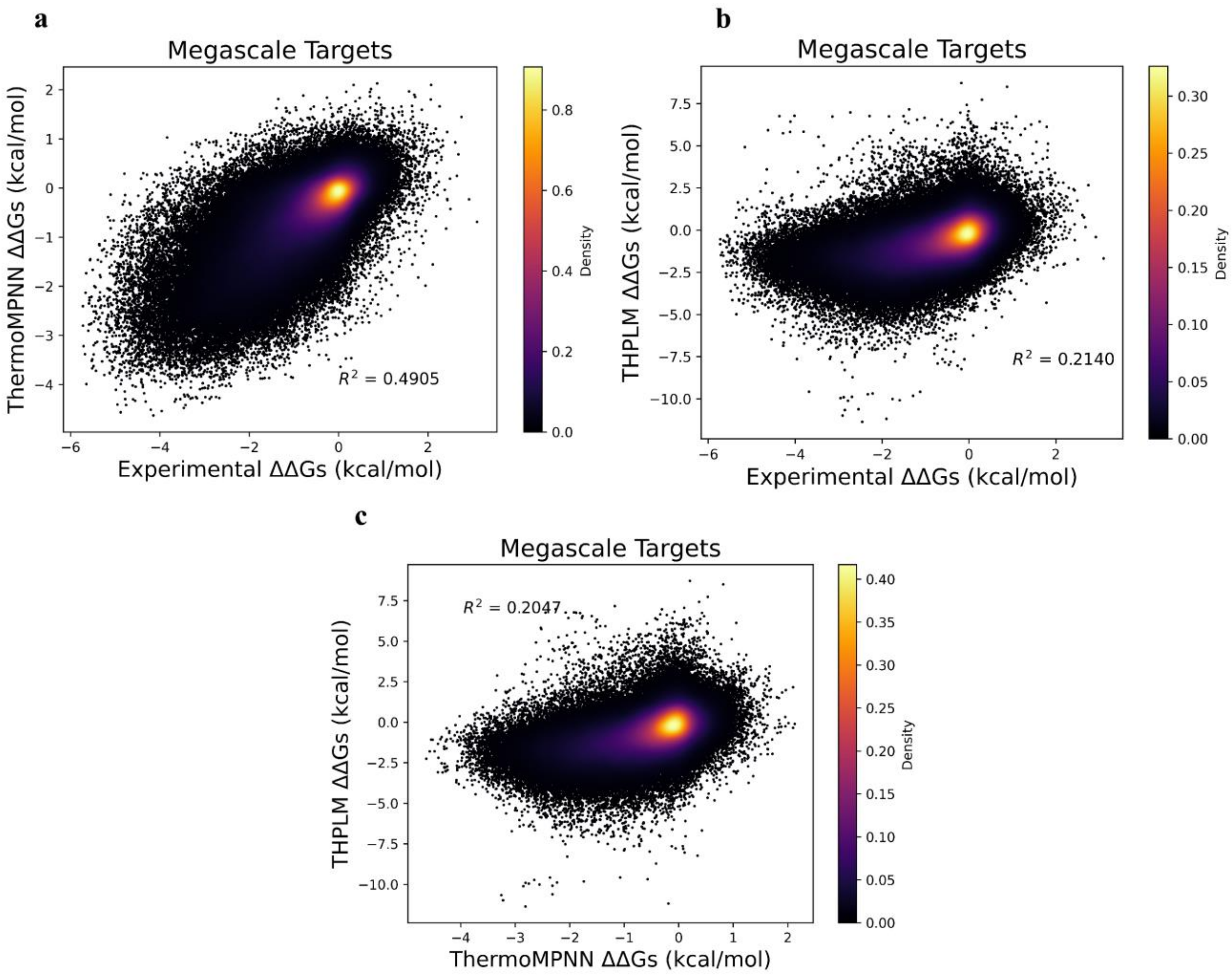

Supplementary Figure 5) Comparison of simulated ΔΔGs from ThermoMPNN and THPLM with experimentally measured ΔΔGs for all available point mutations in the mega-scale set. a) ThermoMPNN-simulated ΔΔGs versus experimental ΔΔGs. b) THPLM-simulated ΔΔGs versus experimental ΔΔGs. c) Comparison of ΔΔGs simulated by ThermoMPNN and THPLM.

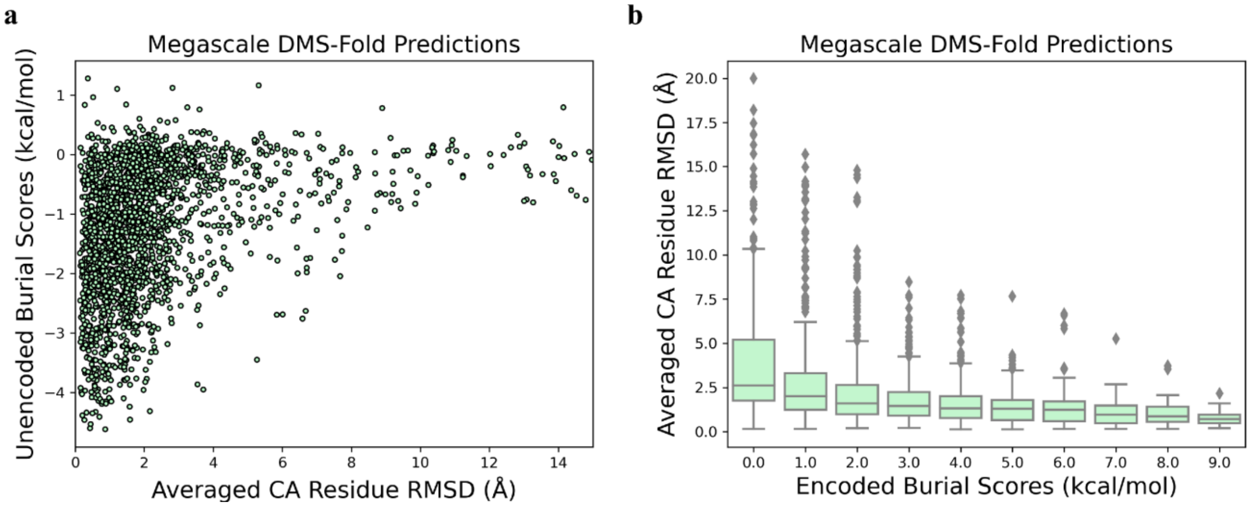

Supplementary Figure 6) Comparison of averaged per-residue α-carbon root-mean-squared deviations (CA RMSDs) of DMS-Fold predicted structures and experimentally derived burial scores of mega-scale proteins. RMSDs were averaged across predictions from 25 seeds a) Scatter plot of residue RMSDs vs unencoded burial scores. b) Boxplots of residue RMSDs for different encoded burial scores.

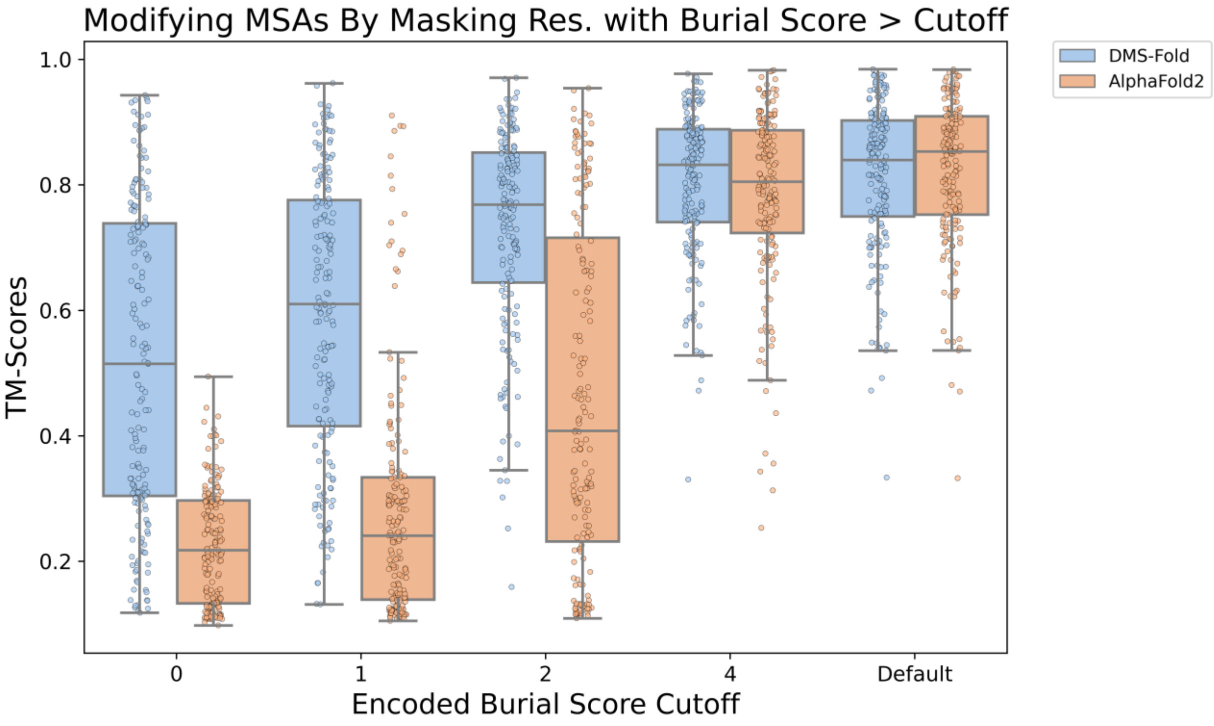

Supplementary Figure 7) Boxplot comparing the TM-Score distributions of DMS-Fold and AlphaFold2 with custom-modified MSAs based off an encoded burial score cutoff. After MSAs were generated for a particular target sequence, these MSAs were modified such that buried residues, meaning residues with an encoded burial score greater than the specified cutoff, were replaced with 'X'. Blue indicates DMS-Fold predictions and orange indicates AlphaFold2 predictions.

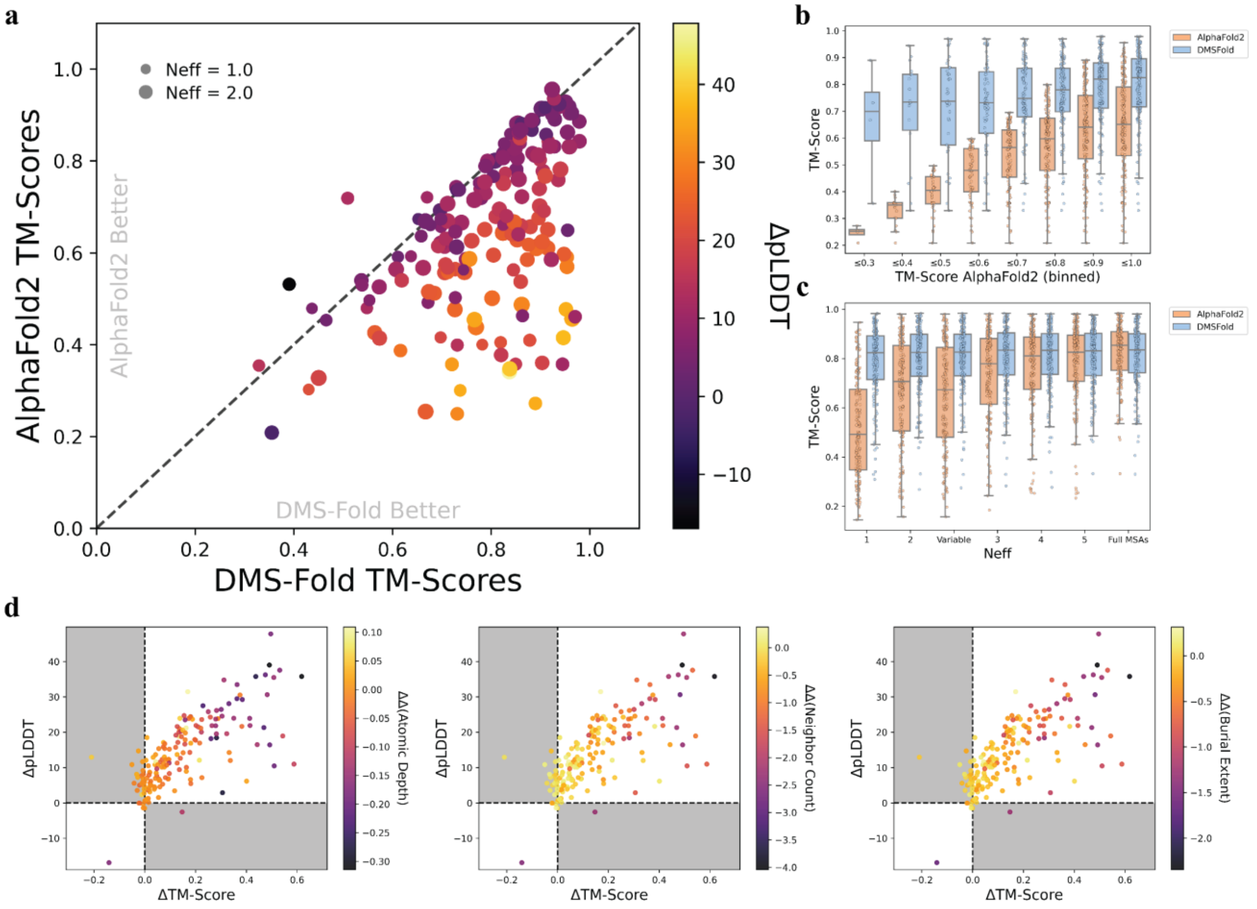

Supplementary Figure 8) Performance of DMS-Fold trained without the Mega-scale proteins, on the Mega-scale set using experimental changes in protein thermodynamic stabilities (ΔΔGs). a) Template modeling score (TM-Score) comparison of predictions from DMS-Fold and AlphaFold2 using a size-dependent number of nonredundant sequences (N_{eff}). Size of each marker represents the N_{eff} used for MSA subsampling. Color represents the change in network confidence, pLDDT between DMS-Fold and AlphaFold2. b) TM-Score distributions of both networks binned to TM-Scores of AlphaFold2 predictions. c) TM-Score distributions of predictions from both DMS-Fold and AlphaFold2 using different uniform N_{eff} values. d) Comparison of changes in pLDDTs and TM-Scores between predictions with DMS-Fold and AlphaFold2. Color represents the change in the difference of solubility metrics for the DMS-Fold structure and the native structure with the AlphaFold2 structure and the native structure.

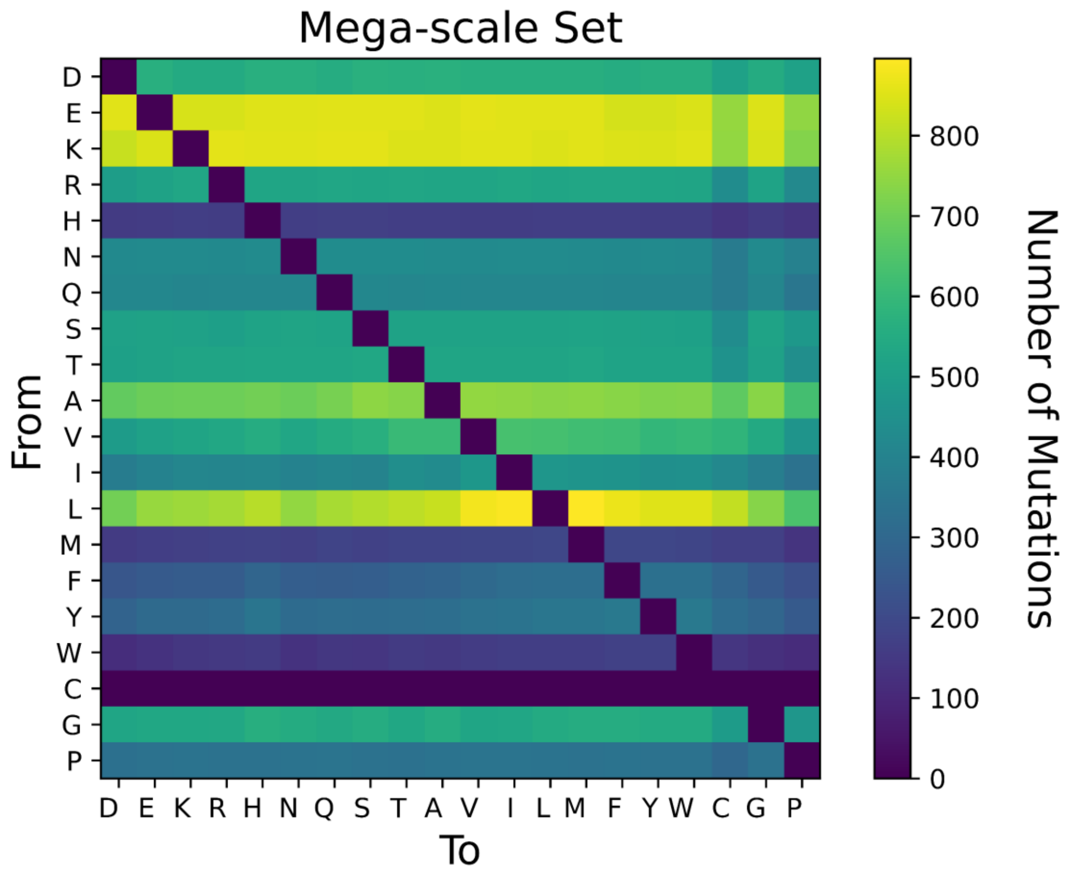

Supplementary Figure 9) Number of mutations across the 175 proteins taken from the mega-scale set. Due to the low number of cysteine residue mutations, these were excluded from our analysis.

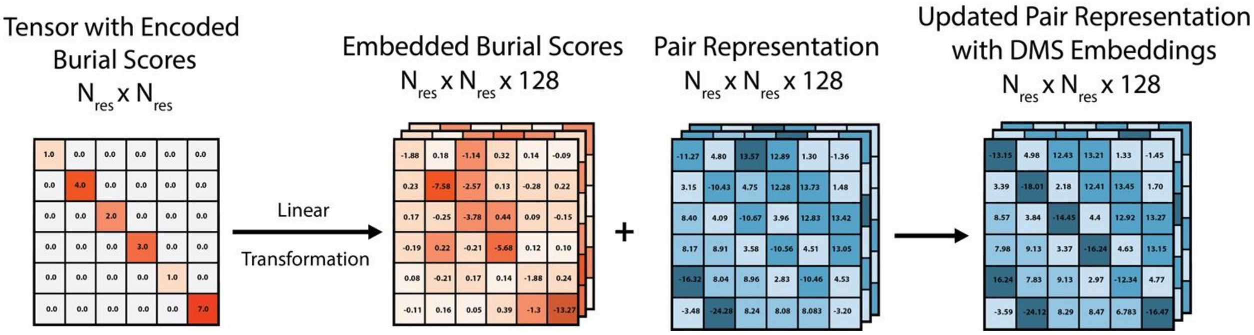

Supplementary Figure 10) Diagram depicting the embedding of the burial scores into DMS-Fold's pair representation. An initial N_{res} x N_{res} is constructed where burial scores are placed along the diagonal. A new linear layer transforms this tensor to match the dimensionality of OpenFold's pair representation, resulting in a N_{res} x N_{res} x 128 tensor. The embedded tensor is then added to the pair representation.

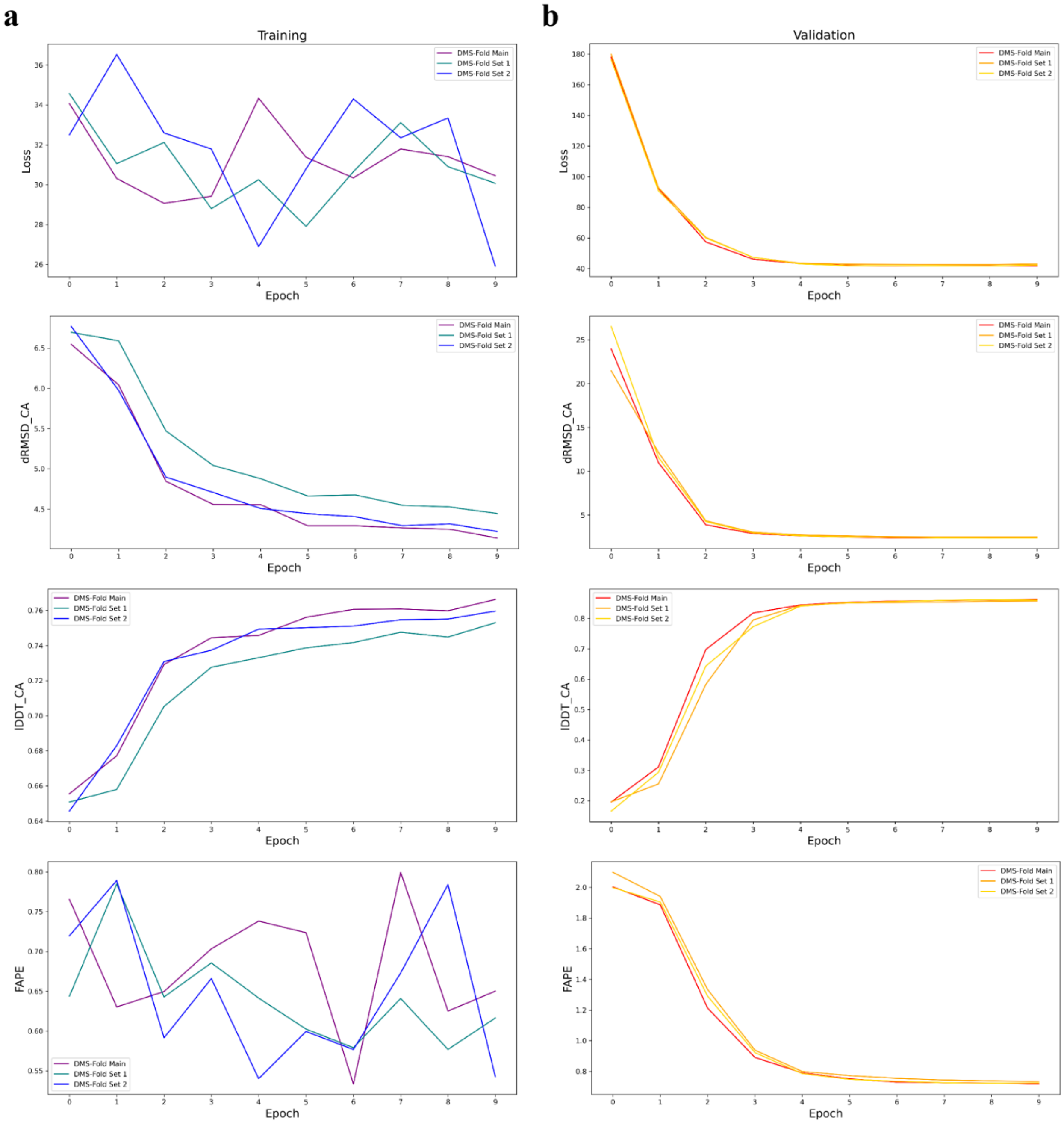

Supplementary Figure 11) Training and validation metrics of DMS-Fold for the main model, mega-scale split with subset 1, and subset 2. a) Subset of DMS-Fold training metrics, including training loss per-epoch, distance root mean squared deviation with respect to the alpha carbon (dRMSD_CA) per-epoch, local distance difference test with respect to the alpha carbon (lDDT_CA) per-epoch, and frame aligned point error (FAPE) loss per-epoch. b) Subset of DMS-Fold validation metrics, including validation loss per-epoch, dRMSD_CA per-epoch, lDDT_CA per-epoch, and FAPE loss per-epoch.

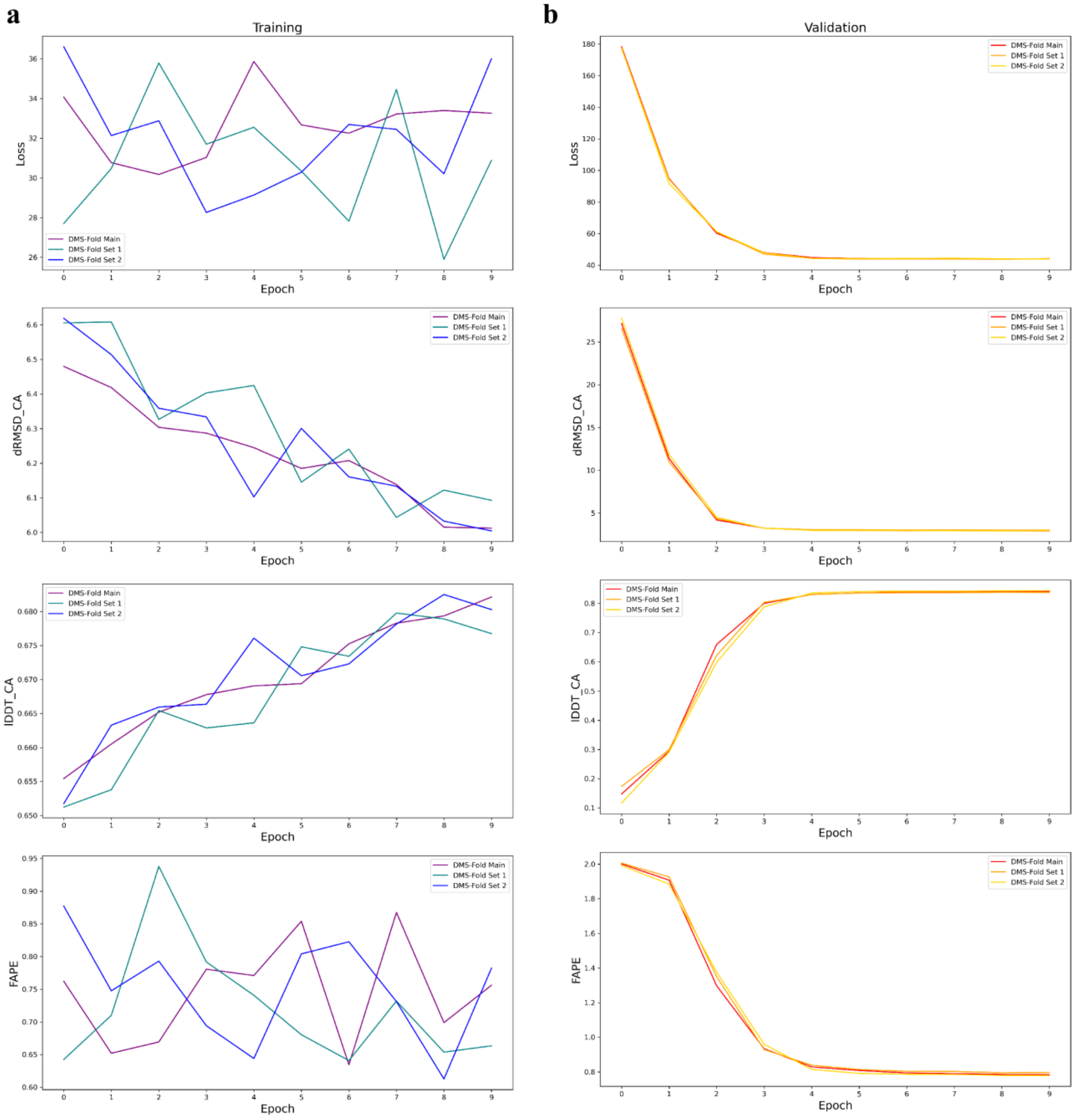

Supplementary Figure 12) Training and validation metrics of DMS-Fold trained using THPLM-derived burial scores for the main model, mega-scale split with subset 1, and subset 2. a) Subset of DMS-Fold training metrics, including training loss per-epoch, distance root mean squared deviation with respect to the alpha carbon (dRMSD_CA) per-epoch, local distance difference test with respect to the alpha carbon (lDDT_CA) per-epoch, and frame aligned point error (FAPE) loss per-epoch. b) Subset of DMS-Fold validation metrics, including validation loss per-epoch, dRMSD_CA per-epoch, lDDT_CA per-epoch, and FAPE loss per-epoch.

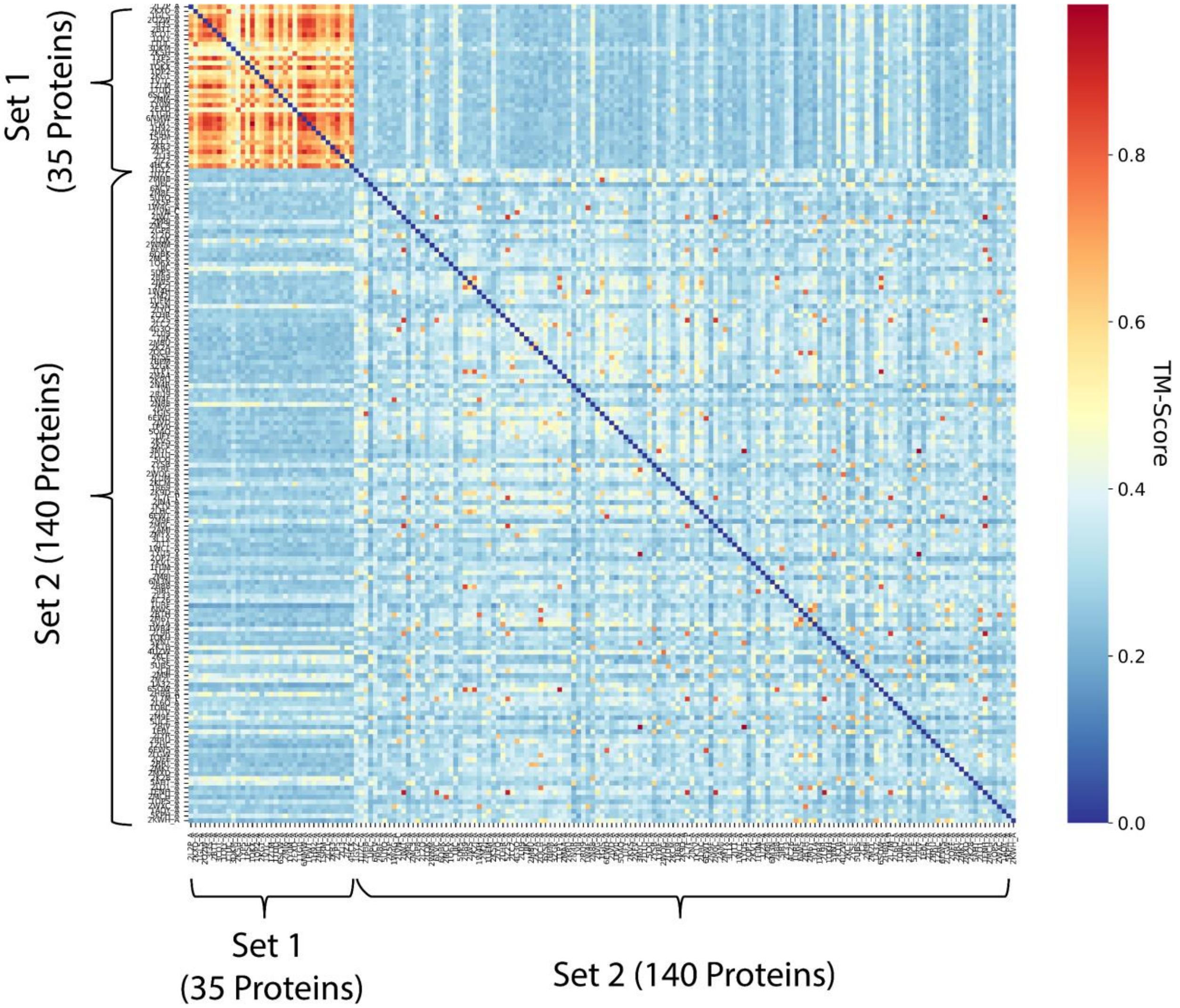

Supplementary Figure 13) Pairwise heatmap of TM-scores between proteins in the mega-scale set. Proteins are grouped and ordered along the axes according to their set designation (Set 1: 35 proteins; Set 2: 140 proteins), with boundaries indicated by black lines. The color scale represents the TM-score, with warmer colors indicating higher structural similarity.

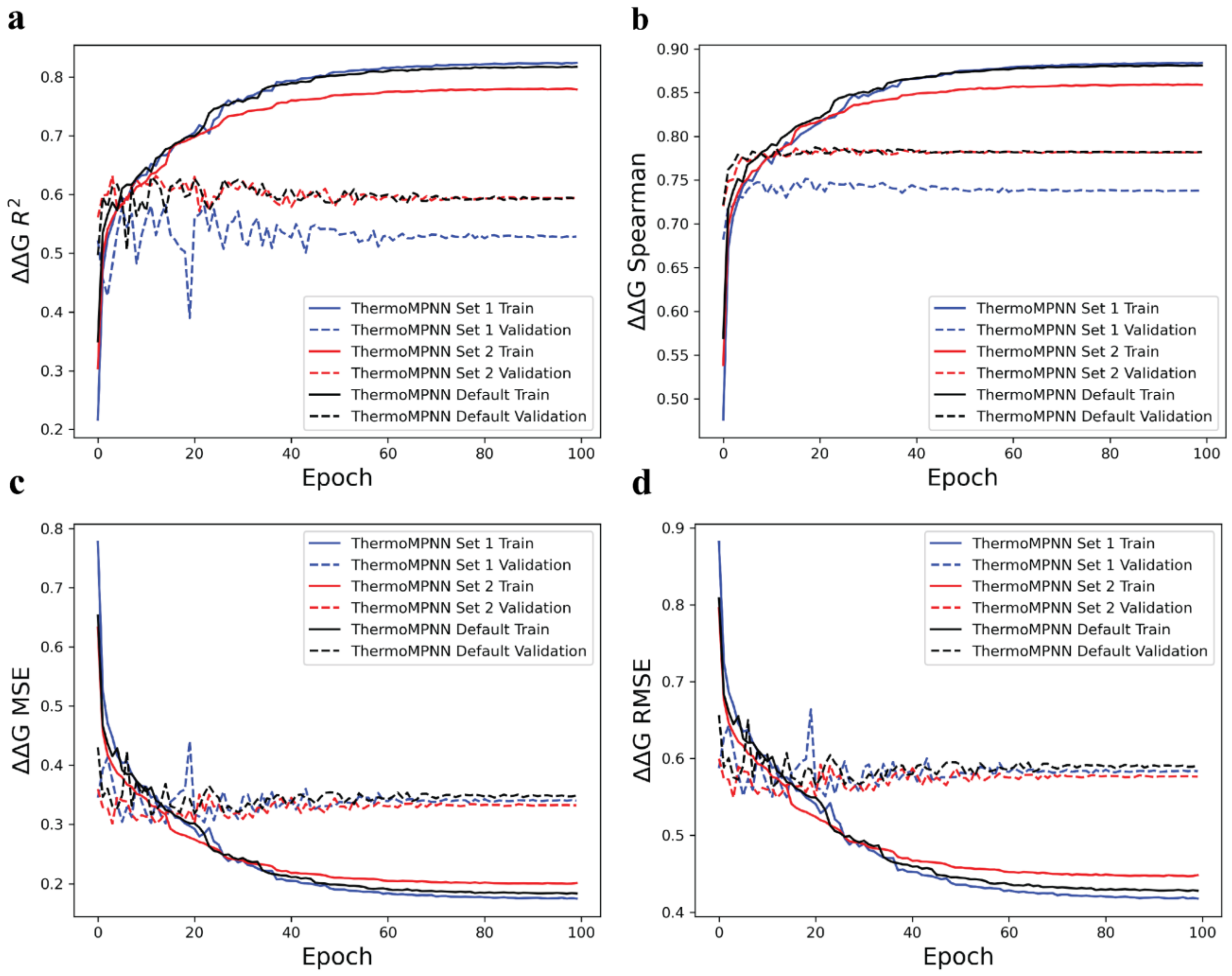

Supplementary Figure 14) Comparisons of the training and validation metrics, shown in black, blue and red respectively, of ThermoMPNN trained with all proteins, proteins from Set 1, and proteins from Set 2, respectively, across 100 epochs. a) Comparison of ΔΔG R^2 metrics during training and validation. b) Comparison of ΔΔG Spearman coefficients. c) Comparison of ΔΔG mean squared error (MSE). d) Comparison of root mean squared error (RMSE).

a

b

c

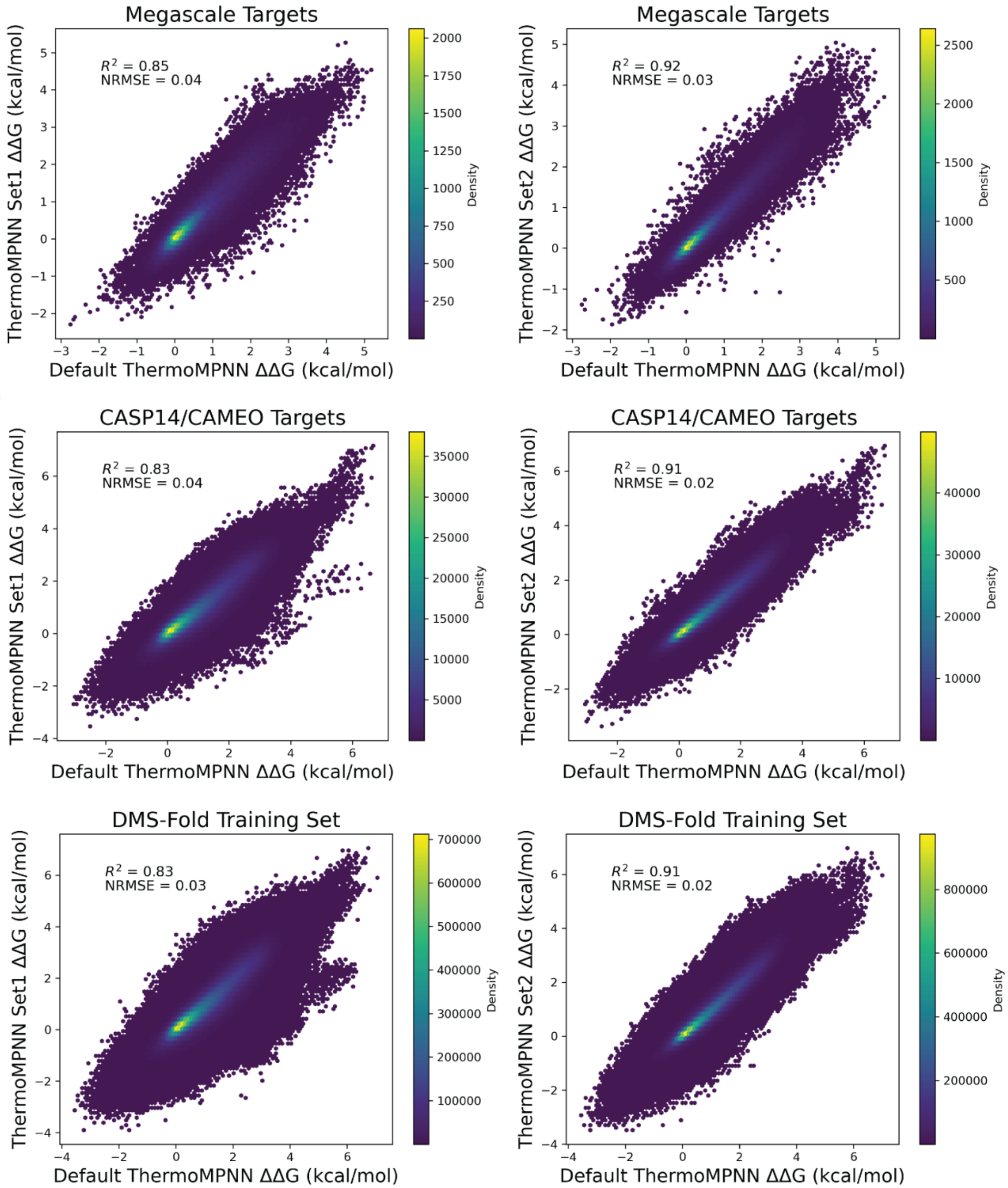

Supplementary Figure 15) Comparison of ΔΔGs from ThermoMPNN using default weights and after training with Set 1 and Set 2 proteins, respectively. Color represents the density of mutation ΔΔGs. a) Comparison of ThermoMPNN ΔΔGs for the mega-scale proteins. b) Comparison of ThermoMPNN ΔΔG for CASP14/CAMEO proteins. c) Comparison of ThermoMPNN ΔΔG of proteins in DMS-Fold's training set (excluding the mega-scale proteins).

Supplementary Table 1) Top 25 mutational types with the highest correlation between mutational stability and burial extent.

Wild Type Residue	Mutated Residue	R^2	Wild Type Residue	Mutated Residue	R^2
**A**	N	0.48	**V**	N	0.41
**A**	K	0.47	**I**	D	0.41
**A**	Q	0.47	**A**	R	0.41
**A**	E	0.45	**I**	Q	0.40
**A**	H	0.45	**I**	E	0.40
**V**	Q	0.44	**V**	K	0.40
**W**	G	0.44	**V**	T	0.39
**V**	E	0.44	**V**	S	0.39
**A**	D	0.44	**I**	N	0.38
**V**	H	0.43	**A**	T	0.38
**V**	D	0.43	**I**	S	0.38
**A**	S	0.43	**I**	H	0.38
**W**	E	0.42			

Supplementary Table 2) PDB IDs of proteins in each subset of the mega-scale set.

Set 1 (35 Proteins)
1GL5, 1H92, 1PSE, 1PWT, 1QKX, 1QLY, 1QP2, 1SRM, 1TG0, 1TUC, 1TUD, 1V1C, 1Y0M, 1YP5, 1ZLM, 2BTT, 2EXD, 2JZ2, 2K5H, 2KGT, 2KR3, 2KXD, 2L2P, 2LCL, 2LJ3, 2LP5, 2M0Y, 2MI6, 2O2W, 3CQT, 3DKM, 3I35, 4HCK, 6NMW, 6SCW
**Set 2 (140 Proteins)**
1A32, 1AOY, 1E0L, 1ENH, 1F0M, 1GJS, 1GYZ, 1I2T, 1I6C, 1IFY, 1JIC, 1K1V, 1LP1, 1O6X, 1OPS, 1ORC, 1PV0, 1QKH, 1R69, 1UFM, 1URF, 1UZC, 1VII, 1W4F, 1W4G, 1W4H, 1WCL, 1WR4, 1YRF, 1YU5, 1ZHC, 2AMI, 2B88, 2B89, 2BTH, 2CJJ, 2D1U, 2GP8, 2HBB, 2JN4, 2JT1, 2JTV, 2JVD, 2JVG, 2JWS, 2JWT, 2K1B, 2K28, 2K2A, 2K5N, 2K5P, 2K9D, 2KCF, 2KCM, 2KFV, 2KRU, 2KVS, 2KVT, 2KWH, 2KZJ, 2L09, 2L2D, 2L33, 2L6Q, 2L7F, 2L7M, 2L9R, 2LC2, 2LGW, 2LHC, 2LHR, 2LO1, 2LQK, 2LUM, 2LVN, 2LYQ, 2LYR, 2M0C, 2M2J, 2M2L, 2M6Y, 2M8E, 2M8I, 2M8J, 2M8U, 2M9E, 2M9F, 2M9I, 2MA4, 2MC5, 2MCH, 2MCK, 2MH8, 2MKY, 2MXD, 2MYX, 2N4R, 2N88, 2OCH, 2OP7, 2QFF, 2RJV, 2RRT, 2RRU, 2RU9, 2WNM, 2WQG, 2WXC, 2YSB, 2YSF, 3L1X, 3MYC, 3V1A, 3ZGK, 4C26, 4G3O, 4UZW, 5AHT, 5JRT, 5KPH, 5LXJ, 5OAO, 5UBS, 5UCE, 5UP5, 5UYO, 5VNT, 5Z2S, 6ACV, 6EWS, 6EWT, 6EWU, 6FVC, 6IWS, 6M3N, 6OBK, 6SOW, 6YSE, 7BPM, 7JJK
